# Prophylactic Incisional Negative Pressure wound therapy (NPWT) for major Amputations (PINTA): protocol for randomized controlled trial of single-use NPWT devices for closed-incision major lower extremity amputations

**DOI:** 10.1093/bjsopen/zraf159

**Published:** 2026-01-01

**Authors:** Megan Power Foley, Ciara Fahey, Anne-Marie Byrne, Roisín Leahy, Laura Dempsey, Daniel Westby, Stewart R Walsh

**Affiliations:** Department of Vascular Surgery, University Hospital Galway, Newcastle Road, Galway, Ireland; Irish Surgical Research Collaborative, Dublin, Ireland; National Surgical Research Support Centre, Royal College of Surgeons Ireland, Dublin, Ireland; National Surgical Research Support Centre, Royal College of Surgeons Ireland, Dublin, Ireland; National Surgical Research Support Centre, Royal College of Surgeons Ireland, Dublin, Ireland; National Surgical Research Support Centre, Royal College of Surgeons Ireland, Dublin, Ireland; Department of Vascular Surgery, University Hospital Galway, Newcastle Road, Galway, Ireland; Department of Vascular Surgery, University Hospital Galway, Newcastle Road, Galway, Ireland; National Surgical Research Support Centre, Royal College of Surgeons Ireland, Dublin, Ireland; Lambe Institute for Translational Research, University of Galway, Galway, Ireland

**Keywords:** surgical complications, peripheral arterial disease, diabetes mellitus, medical device

## Abstract

**Background:**

Major lower extremity amputations are frequently performed for end-stage peripheral arterial disease and progressive diabetic foot complications. Wound complications after amputation affect up to one-third of limbs. The patient cohort undergoing amputation are typically high risk for poor wound healing, often with unmodifiable risk factors in an urgent clinical setting. Incisional negative pressure wound therapy (NPWT) has been shown to reduce wound complications in other high-risk populations. This randomized controlled trial investigates whether prophylactic NPWT reduces wound complications in patients after major amputation compared with standard dry dressings.

**Methods:**

This protocol describes a prospective, multicentre, randomized controlled trial with an internal pilot recruiting patients undergoing major lower extremity amputation for any indication. Limbs will be randomized to receive either a single-use NPWT device on their closed surgical incision or a dry dressing. The primary clinical outcome is the rate of wound complications. Secondary outcomes include reoperation rates, length of hospital stay, cost-effectiveness of NPWT, and patient-reported quality of life. Follow-up will continue to 6 months after surgery. The initial pilot phase has a recruitment target of 96 limbs, whereas an estimated 728 patients will be required to power a definitive trial adequately.

**Discussion:**

This trial aims to supplement the existing poor-quality data on this important aspect of care and equip healthcare professionals to make cost-effective decisions regarding postoperative wound management.

## Teaser

Protocol for a pragmatic randomized controlled trial comparing prophylactic negative pressure wound therapy devices with standard care for closed-incision major lower extremity amputations. Outcomes include composite wound complications at 30 days, patient-reported outcomes on quality of life and wound-related quality of life, and cost-effectiveness. Adequately powered to 90% with a target sample size of 728 patients.

## Background

Major lower extremity amputations are frequently performed for unreconstructable peripheral arterial disease and progressive diabetic foot complications^1,2^. As the population ages and the prevalence of diabetes increases, rates of diabetic-related major amputations are projected to increase despite advancing revascularization techniques. In both the UK and Ireland, rates of major amputation are significantly higher in the diabetic population^3,4^. In the United States, approximately 185 000 major amputations are performed annually, and the number of patients living with limb loss is projected to double from 1.5 million to 3.6 million by 2050^5^. Up to one-third of patients experience perioperative wound complications after major amputation^6–10^. Conversion from below-knee amputation (BKA) to above-knee amputation (AKA) is estimated to occur in 12–34% of limbs, with wound infection causative in a significant number of cases^8^. The 30-day readmission rate after a lower extremity amputation ranges from 29 to 46%^9,11^. Prolonged inpatient stays, multiple procedures, costly comorbidities, and high rates of readmissions lead to patients with peripheral arterial disease accumulating hospital costs six times those of general inpatients^12^. Similarly, the healthcare costs and resource utilization of diabetic foot care in England is greater than the combined cost of breast, prostate, and lung cancers^13^.

Many factors influence the risk of wound complications^14–18^. Notably, the presence of proximal occlusive arterial disease is a major influence on stump healing. Patient factors such as smoking, diabetes, obesity, malnutrition, and chronic kidney disease are non-modifiable, particularly in the emergency setting. However, surgical factors may be altered in an effort to reduce the risk of wound complications. One variable amenable to alteration is what dressing is applied to the closed incision upon procedure completion. The type of dressing may influence factors such as bacterial access to the wound, the development of collections of blood or fluid within the wound bed, or discharging onto the wound edges. Collectively, these wound factors increase the risk of wound infection. Therefore, dressings that reduce these factors have the potential to reduce wound breakdown, thereby reducing the burden for patients and healthcare systems.

## Trial rationale

Negative pressure wound therapy (NPWT) has been used successfully to treat a variety of complex wounds^19^. The technology has been adapted into single-use battery-powered dressings designed for closed surgical incisions rather than cavitating, open wounds. These NPWT dressings consistent of an open-cell solid foam, placed on top of the incision and then covered with a semipermeable membrane. A sealed tube connects the foam to a pump to create a partial vacuum over the wound. This negative pressure leads to a sealed environment that prevents bacterial migration into the wound while removing blood and serous exudate. The systems can be applied and, once sealed, left in place for up to 7 days. Single-use NPWT systems (Smith & Nephew’s PICO™ and Acelity’s Prevena™) are now widely available.

Prophylactic NPWT in the setting of closed surgical incisions has been widely evaluated. A recently updated Cochrane review (2022) analysed data from 62 randomized controlled trials (RCTs) and six economic studies, including 13 340 participants across a range of surgical specialities (gastrointestinal, gynaecological, vascular, orthopaedic, and cardiothoracic)^19^. The review was concluded with moderate certainty that prophylactic NPWT reduces the incidence of surgical site infection (SSI) though not wound dehiscence. Nonetheless, it should be noted that the definition of SSI varied amongst included studies. As such, it remains unclear whether NPWT reduces all wound infections, just superficial infections alone, or deep infections.

Stemming from this first success, the role of NPWT has been investigated across specialities and anatomical locations. In vascular surgery, the majority of the literature has focused on vascular groin exposures, an area known to be high risk for wound infection and breakdown due to a constellation of patient and wound factors. A meta-analysis^20^ of eight RCTs with 1125 patients reported an overall reduction in SSI rates but no difference in wound dehiscence, seroma formation, and reoperation and readmission rates. Lower extremity amputations are another wound bed vulnerable to deterioration; however, the literature^21^ investigating the impact of incisional NPWT on amputations is limited and underpowered.

To answer these outstanding questions, a multicentre RCT (“PINTA”) is proposed comparing prophylactic single-use NPWT with standard dressings in patients with a closed incision following an lower extremity amputations.

## Objectives

The single main research question for this trial is as follows: in adult patients with major amputations, do single-use NPWT devices reduce post-operative wound complications?

### Primary objective

The primary objective for the trial is to quantify and analyse differences in the rates of composite wound complications at 30 days after amputation between NPWT and standard dressings. Wound complications are defined as infection, either deep or superficial; wound dehiscence, either partial (fascia intact) or complete (fascia breached); seroma; haematoma; and wound necrosis.

### Secondary objectives

The secondary objectives comparing standard dressings with NPWT dressings are as follows:

- To determine and analyse any difference in SSI rates;
- To determine and analyse any difference in wound dehiscence rates;
- To determine and analyse any difference in length of stay from surgery to discharge from acute hospital, not including subsequent days spent in step-down care of an equivalent setting;
- To determine and analyse any difference in readmission rates for wound-related complications in the 6 months following major amputation;
- To quantify and analyse differences in patient-reported general health-related quality of life in the 6 months following major amputation;
- To quantify and analyse differences in patient-reported wound-related quality of life in the 6 months following major amputation;
- To quantify and analyse differences in patient financial impact in the 6 months following major amputation;
- To determine the number and nature of further interventions related to amputation in the first 6 months following the index procedure;
- To investigate resource use and thereby cost-effectiveness of single-use, prophylactic NPWT devices *versus* standard dressings for closed-incision amputation wounds.

## Trial design and endpoints

### Statement of design

**Fig. 1:**
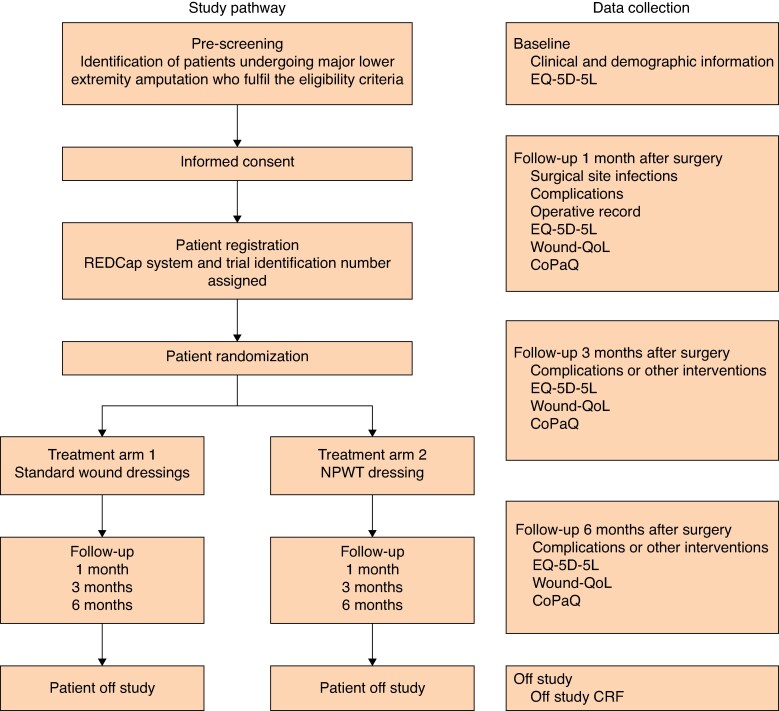
PINTA study pathway including treatment arms and follow-up intervals NPWT, negative pressure wound therapy; EQ-5D-5L, patient quality of life questionnaire; Wound-QoL, patient chronic wound quality of life questionnaire; CoPaQ, cost for patients questionnaire; CRF, case report form.

This is a prospective randomized controlled, assessor-blinded trial, with participants allocated to one of two parallel arms in a 1 : 1 fashion. The primary trial centre is University College Hospital Galway, Ireland, and other Irish centres being evaluated include University Hospital Limerick, University Hospital Waterford and, in Dublin, St James University Hospital, St Vincent’s University Hospital, Tallaght University Hospital, and Beaumont Hospital. All adult patients presenting at trial centres in an inpatient or outpatient capacity requiring a lowet extremity amputation suitable for primary closure are potentially eligible for inclusion. The study pathway is outlined in *Fig. 1*.

The trial will be conducted at Irish tertiary vascular centres over a period of 3 years. Participating patients will have a clinical follow-up at the centre’s surgical outpatients department for a minimum of 6 months. This is standard clinical practice for all specialties following these types of procedures. At 1, 3, and 6 months after amputation, patients will complete the quality of life (EQ-5D-5L^TM^), chronic wound quality of life (Wound-QoL^TM^), and cost for patients (CoPaQ^TM^) questionnaires to evaluate the functional and financial impact of amputation and amputation-related complications on the patients. Late complications and surgical interventions related to their index procedure will also be collected to evaluate the financial impact on the healthcare system.

### Outcomes

#### Primary efficacy outcome

The primary outcome measure is any wound complication of the amputation stump, including SSI, dehiscence, seroma, haematoma, and stump necrosis. The Centre for Disease Control and Prevention^14^ definitions of SSI will be used (*Appendix 1*). As part of trial on-boarding, the Centres for Disease Control and Prevention (CDC) criteria for SSI will be shared to all clinicians involved in patient assessment. The treating clinical team will make the diagnosis of wound infection, as per routine clinical practice. Given that prompt diagnosis and treatment of wound infection is a core part of routine clinical care, the treating clinicians will typically document such a management change in the patients’ medical records. In addition, an independent outcome assessor will review data collected in the clinical report forms to confirm the diagnosis of a wound complication. For the purposes of the trial, any wound infection requiring ongoing medical intervention or that has led to reoperation at or after 30-day review will be considered a deep infection. Furthermore, any dehiscence involving the fascia or requiring interposition vacuum-assisted closure (VAC) therapy will be considered a deep dehiscence. Similarly, any stump necrosis involving the fascia or deeper structures will be considered deep necrosis, whereas superficial necrosis will be confined to the skin and subcutaneous tissues.

#### Primary safety outcome

The primary safety endpoints in the intervention group are any complications arising from the additional use of NPWT devices and the requirement for subsequent acute or subacute reintervention (at any time) as a result of the primary intervention.

#### Secondary outcomes

The secondary outcome measures comparing standard dressings to NPWT dressings in this trial are:

- Number of recruited patients within the internal pilot will be assessed;
- Number of all wound complications (composite) in each arm will be collected;
- Number of SSIs in each arm will be assessed: any SSI meeting the CDC criteria will be documented and described as superficial or deep;
- Number of wound dehiscence rates in each arm within 6 months of index surgery will be assessed: any wound dehiscence will be documented and described as superficial or deep;
- Number of days to discharge for each arm will be assessed: the length of time the patient is in hospital from date of surgery to both the date of discharge and the date that the patient is deemed medically fit for discharge will be recorded;
- Number of reoperations within 6 months of index surgery in each arm will be assessed: any reoperations undertaken will be recorded;
- Number of readmissions to hospital within 6 months of index surgery in each arm will be assessed: all readmissions to hospital related to the surgical procedure will be recorded;
- Impact on quality of life: the EuroQoL EQ-5D-5L questionnaire is a validated measure of health-related quality of life. It comprises a five-dimension health status classification system and a separate visual analogue scale;
- Differences in wound-related quality of life in the 6 months following major amputation will be captured using the Wound-QoL questionnaire;
- Economic impact: the financial impact of patients’ out-of-pocket expenses will be recorded using the validated CoPaQ questionnaire. Patient self-reported information on service use has been shown to be accurate^11^. Healthcare resource use will be recorded for the economic analysis. Unit cost data will be obtained from national databases where available but where not available the unit cost will be estimated in consultation with the hospital finance department;
- Resource use and cost-effectiveness data, including number of NPWT devices utilized and troubleshooting with NPWT devices.

## Trial population

### Inclusion criteria

Adult patients, aged 18 and over, with capacity to consent undergoing lower extremity amputation, including BKA, through-knee amputation, and AKA, for any indication, suitable for primary closure of the surgical incision using either interrupted or continuous sutures.

### Exclusion criteria

- Women who are pregnant and/or breastfeeding;
- Amputations performed without primary skin closure, including guillotine amputations, amputations deliberately left open for drainage purposes, and amputations with soft tissues defects at the stump;
- Clinically absent femoral pulse.

### Recruitment and informed consent

#### Informed consent of the inpatient

Patients will be screened from clinic and operating lists in trial centres. All patients listed for a major amputation will be assessed for eligibility. Screening logs will be maintained at each site to record the number of patients assessed for eligibility and the reasons for any exclusions. Those patients who decide they do not wish to participate or withdraw from the study will be given the opportunity to discuss with the research team the reasoning behind the decision to not take part.

The process of obtaining informed consent will be conducted in compliance with the principles of good clinical practice and requirements of the approving research ethics committee. Patients will be approached for consent before they have the operation. Consent to enter the trial will be sought from each subject only after a full written and verbal explanation has been given and, insofar as is possible, time allowed for consideration. Patients will be provided the opportunity to ask questions and discuss the study with the research team. Patients will be given an information leaflet (*supplemental data 1*). As a reasonable percentage of major amputations are performed as urgent or emergent procedures, in these circumstances the available time for consideration will be truncated. The consent process will be conducted by the trial investigators in all cases. All original signed consent forms will be kept in the investigator study-specific site file. There will be three copies of each consent form: one for the patient’s medical record, one for the patient, and one for the study team.

#### Informed consent of the outpatient

The minority of patients undergoing major amputation may come to a shared decision in the outpatient setting and then return at a separate date for elective admission. Eligible patients will be given an information sheet at their outpatient visit and the trial will be explained to them by a team member. Written informed consent will be obtained immediately before the procedure by an investigator.

### Randomization

Following the consent process, eligible patients will be enrolled in the study through the online randomization system at the time of surgery in the operating theatre. Randomization will be on a one-to-one basis using a computer randomization program, with balanced allocation of patients across the two treatment groups, stratified by trial centre. All monitoring operating theatres can provide immediate internet access permitting the use of a secure 24-hour web-based randomization system to generate treatment allocation during surgery. Final eligibility will be confirmed by the treating surgeon at the end of the operative procedure before the wound dressing is applied, as a small subset of patients may be deemed clinically inappropriate for wound closure. A unique trial number will be assigned to each individual at the time of randomization. Patients will be randomized to one of two groups. There will be no sham intervention.

Group 1: single-use NPWT device applied to the closed skin incision after amputation.

Group 2: standard dry dressing applied to the closed skin incision after amputation.

### Baseline patient data

Patients will all have a full medical history taken and clinical examination as part of their standard care. Furthermore, a EQ-5D-5L quality of life questionnaire will be completed. The following baseline clinical and demographic data will be recorded:

- Sex;
- Height;
- Weight;
- Year of birth;
- Smoking status;
- Diabetes;
- Peripheral arterial disease (Ankle Brachial Pressure Index < 0.6);
- Previous revascularization ipsilateral leg;
- Surgical speciality performing amputation;
- Rockwood Clinical Frailty Score (> 60 years old only).

### Internal pilot phase data

- Number of eligible patients;
- Number of consenting patients;
- Number of patients declining participation;
- Assessment of the number of patients who attend or miss scheduled follow-up;
- Reasons for missed follow-up appointments;
- Barriers to timely scheduling of interventions and follow-up.

### Interventions

#### NPWT

Negative pressure dressings use an open-cell foam laid on top of the wound, which is then covered with an impermeable adhesive membrane. A sealed tube punctures the membrane to connect the dressing in-built pump, leading to the creation of a partial vacuum over the wound. The device is licensed to stay *in situ* and sealed for 5–7 days. In some cases, depending upon the treating surgeon’s normal practice and the volume of wound exudate, the wound may be redressed again on the ward. The postoperative day that the dressing was first removed will be recorded, as will the application of any subsequent NPWT dressings or dry dressings. For the purpose of this trial, NPWT device(s) may be used for up to 14 days after surgery.

#### Standard dressing

Standard dressing for an amputation wound comprises a non-adhesive layer applied directly to the wound, covered by a sealed dressing, supplemented with protective padding. Such standard dressings do not use negative pressure. The precise details of the materials used will be at the discretion of the treating surgeon as per their routine practice but the details of each dressing applied in the trial will be noted. The duration that each dry dressing is left *in situ* varies between surgeons, though the majority will leave the first dressing for a minimum of 48 hours and a maximum of 5 days. For the purpose of this trial, the type and duration of control dressing will be recorded, including the postoperative day the dressing was first removed.

Of note, it is some surgeons’ usual practice to use a drain in the amputation stump delivered out through a separate incision to protect against haematoma formation; the drain is usually ready for removal at 48–72 hours after surgery. There is a lack of high-quality evidence supporting drain use after major amputation; however, it a widespread practice. Achieving a durable seal for NPWT around a drain can be technically challenging, and a seal may be lost once the drain is removed; extra care should be taken to maintain a seal once the drain is removed. For the purpose of this study, surgeons should not alter their regular practice regarding drain use. The presence or absence of a wound drain will be recorded in the procedural data, allowing for potential subanalysis of the impact of drain use on outcomes.

### Schedule of events

#### Follow-up

At 1, 3, and 6 months, participants will be followed up in clinic where Wound-QoL, CoPaQ, and EQ-5D-5L quality of life questionnaires will be collected (*Fig. 1*). Details of late complications and any further surgical interventions will be recorded at these time points also. A window of +/− 2 weeks is permitted for each time point. These data will be uploaded onto the secure REDCap database. The number and timing of any subsequent follow-up appointments for clinical purposes will be at the discretion of the treating surgeon.

### Device assessment

The single-use NPWT device will be reviewed daily by the surgical team and issues with sealing and exudate will be recorded. The number of devices left *in situ* sealed appropriately for the recommended period (minimum 5 and maximum 7 days) will be noted. All episodes of resealing on the ward will be documented. Reasons for premature removal of the NPWT device will be recorded.

#### Wound assessment

The surgical incision will be reviewed by the clinical team as appropriate while the patient remains in hospital. All incidences of wound complications will be documented prospectively. The presence of SSI will be determined using the CDC criteria. The time taken in days from surgery to wound healing will be recorded. At 30 days, the surgical incision will be reviewed by a surgeon blinded to the initial wound management strategy and categorized as ‘healed’ or ‘not healed’.

### EQ-5D questionnaire

Quality of life will be evaluated by completion of the EQ-5D form at 3 and 6 months. These data will be further utilized to generate health economic data.

### Crossovers

Any crossovers between treatment groups shall be highlighted and reported in the final report. Participants’ follow-up data will continued to be recorded on an intention-to-treat basis, as such patients will continue to be analysed in the group they were originally randomized to whether they received the correct intervention or not. Reasons for protocol deviation will be documented in the site logs, for example NPWT device unavailable or unable to achieve seal despite multiple attempts or surgeon preference. Patients in the control group with unhealed wounds after the initial 14-day review period for whom the treating surgeon feels may benefit from application of a single-use NPWT device will be offered one.

### Withdrawals during follow-up

Participants may decide to discontinue the participation in the trial at any time without prejudice. Such a decision to withdraw will not affect the standard of care the patient receives. They may either withdraw from completing any further questionnaires but allow the research team to collect relevant hospital data in relation to postoperative complications, which are recorded as part of normal standard of care, or they may withdraw completely from the study. Data obtained up into the point of withdrawal will be included in the final analysis; thereafter, no further data will be collected for the participant. Patients will be followed up as per standard of care should they withdraw. The principal investigator may withdraw an individual from the trial should they be subject to an adverse event whereby it is in the patient’s best interest to be withdrawn or should they meet any of the exclusion criteria.

### Loss to follow-up

Prior to consent, participants will be educated as to the importance and timing of the follow-up protocol. Should follow-up appointments be missed, investigators will endeavour to contact patients by phone or via community liaisons to ensure timely follow-up within the trial protocol. All losses to follow-up and its cause shall be recorded and reported in any trial data.

### Protocol violations

Patients may be excluded in the phase after randomization if it is established they were unable to adhere to trial procedures, for example completing questionnaires. Patients can also be withdrawn at the principal investigator’s discretion if it is in the best interests of the patient.

### Premature termination of the study

The trial may be temporarily suspended or prematurely terminated if there is sufficient reasonable cause. Written notification, documenting the reason for trial suspension or termination will be provided to the regional ethical committee and all trial investigators by the principal investigator.

Circumstances that may warrant termination or suspension include, but are not limited to:

- Determination of unexpected, significant, or unacceptable risk to participants;
- Demonstration of efficacy that would warrant stopping;
- Insufficient compliance to protocol requirements;
- Data that are not sufficiently complete and/or evaluable;
- Determination of futility.

## Safety parameters

### Potential adverse events related to intervention

Primary safety endpoints include adverse events related to the use of single-use NPWT devices. The risk of any significant adverse event occurring is deemed unlikely; however, all reasonable measures to avoid these events will be undertaken. Any adverse events will be recorded and reported in any trial data, namely local reactions to the NPWT device.

### Definition of serious adverse event

Any event that results in death, a life-threatening adverse event, inpatient hospitalization or prolongation of existing hospitalization, a persistent or significant incapacity or substantial disruption of the ability to conduct normal life functions, or a congenital anomaly/birth defect is deemed a serious adverse event.

### Event reporting

Any safety concerns arising with the used of the NPWT dressings should be reported to the Health Protection Regulatory Authority via the online reporting system https://www.hpra.ie/homepage/about-us/report-an-issue/mdiur or by email to devices@hpra.ie. The sponsor should be notified. Any local hospital procedures in place for reporting safety concerns should be followed.

### Trial audit

Regular audit of trial conduct shall be undertaken throughout the study period. The Trial Steering Committee shall convene 6 monthly to review trial proceedings internally and address any methodological, clinical, and ethical concerns. External review shall be undertaken annually by the local research ethics committee, with further data monitoring from an independent research advisor from the National University of Ireland, Galway.

## Statistical considerations

### Statistical analysis

Once randomization and intervention is complete, patient outcomes will be assessed using an intention-to-treat analysis. All losses to follow-up at any time point will be recorded and reported. A blinded biomedical statistician will conduct all analyses independently of the data collection team. Statistical analysis will be conducted using SPSS^TM^ Version 29 (IBM Corp, Aramonk, New York, United States). Baseline data will be summarized to check comparability between treatment arms. The main analysis will investigate differences in the primary outcome measure, the proportion of patients with any wound complication at 30 days after major amputation.

Use of randomization by stratification should ensure balance in the recruiting centre in both treatment groups. Whereas clustering effects are not expected to be important for this study, in reality the data may be hierarchical, with patients naturally clustered into groups by recruiting centre. This will be accounted for by generalizing the conventional linear (fixed-effects) regression approach to a mixed-effects logistic regression analysis. This model will be used to assess differences in wound complication rates between the study intervention groups, with results presented as odds ratios with associated 95% confidence intervals. The mixed-effects model will include a random effect to account for any heterogeneity in response due to the recruitment centre and fixed effects to adjust for vascular status. An identically structured and formulated mixed-effects linear regression model will be used to assess the effects of the interventions on secondary outcomes and EQ-5D-5L (at 1, 3, and 6 months) that, for the purposes of analysis, will be assumed to be approximately normally distributed. Other dichotomous outcome variables, such as complications related to the trial interventions, will be analysed in the same manner as the primary outcome. Temporal patterns of any complications will be presented graphically, and, if appropriate, a time-to-event analysis (Kaplan–Meier survival analysis) will be used to assess the overall risk and risk within individual classes of complications. All reported tests will be two-sided and considered to provide evidence for a significant difference if *P*-values are less than 0.05 (5% significance level. Results from this trial will also be compared with results from other trials and reported in accordance with CONSORT guidelines. Whereas there are a multiplicity of secondary outcomes, considering CONSORT guidelines and regulatory body preferences, the authors do not plan to adjust for type 1 errors due to the high risk of a corresponding increase in type 2 errors^22^.

### Sample size

The data on the impact of prophylactic NPWT on wound complication following major amputation are limited. Retrospective cohort studies^6–10^ of wound complications with standard therapy after amputation reportedly range from 10 to 34%. In a recent meta-analysis^21^ of NPWT for major amputations, the pooled proportion of composite wound complications for patients using NPWT was 16%. For planning purposes, and based on the previously published range, it has been assumed that 25% of participants randomized to standard dressings will develop a wound complication. To investigate an absolute risk reduction of 10% with the use of NPWT (that is, a wound complication rate of 15%), with 90% power at the 5% significance level, a minimum of 331 patients will be required in each arm. To allow for losses to follow-up and withdrawals, 10% will be added to each arm. Therefore, the total projected sample size for the full trial is 728 patients.

## Ethical considerations

### Ethical approval

Ethical approval has been approved centrally by the Galway Clinical Research Ethics Committee, Galway, Ireland. The ethics committee reference is C.A. 3077 (*supplemental data 2*). Ethics are in process in seven other Irish vascular centres. Of note, recruitment shall not commence at any other centre until full ethical approval has been confirmed with the local ethical committee. The trial was registered in ClinicalTrials.gov (NCT06025253) on 4th December 2023.

### Data protection

All data shall be managed in the strictest confidence by approved trial investigators in accordance with Irish data protection law. Data will be entered electronically by authorized site personnel into a secure electronic data capture system (REDCap), which is GDPR, FISMA, and HPAA compliant. Each participant will be assigned a trial identification number at their recruiting hospital for use in the trial database. No patient identifiable information will be entered. A paper-based patient identification log and patient consent log will be securely maintained at each recruiting site in a locked filing cabinet with restricted access. The logs containing patient identifiable information will not leave the patients’ treating hospital. The investigator must maintain accurate documentation (source data) that supports the information entered in the REDCap database. The investigator is responsible for verifying that data entries are accurate and correct. Direct access to source data/documents will be required for trial-related monitoring and/or audit by the sponsor, health service executive, or regulatory authorities as required. Paper and electronic data will be retained for 10 years following completion of the trial.

## Discussion

As rates of major lower extremity amputations persist, strategies to reduce wound-related complications in this high-risk patient cohort are needed. Impaired wound healing prolongs hospitalization, increases healthcare-related costs, negatively impacts patient’s psychological well-being, and delays progress with prosthetic rehabilitation^23^. The use of prophylactic incisional NPWT after laparotomy has been thoroughly researched and found to reduce rates of SSIs significantly, in particular in obese patients or patients undergoing emergency surgery^24–26^. Based on these promising results, incisional NPWT has been applied to multiple other surgical sites, both in emergency and elective settings. It is widely accepted that the most benefit is seen with wounds at high risk of infection and breakdown, due to both local and patient factors, rather than ‘clean’ elective surgeries in robust patients. Amputations make for an interesting cohort, where the majority of incisions are ‘clean’ in the traditional sense—without faecal or environmental contamination—but are affected by dysvascular wound beds and comorbid patients.

The application of NPWT on the lower extremity is limited. Studies specifically investigating the impact of incisional NPWT on closed-incision major amputations are scarce. Preceding the use of ready-made topical NPWT devices, Zayan *et al*.^27^ published a retrospective case series using standard VAC foam dressings on closed incisions on seventeen primary and eight revision amputations. During initial follow-up, they reported one SSI treated with oral antibiotics and no dehiscence, seroma, or haematoma. An early meta-analysis^28^ of three retrospective case-control studies including 457 patients reported a significant association between incisional NPWT and fewer postoperative complications after major amputations. However, this was composite outcome not specific to wound complications and all of the included studies were of moderate-to-serious risk of bias. A subsequent meta-analysis^21^ of nine studies, including three small, underpowered RCTs and six retrospective comparative studies, reported a significant association between prophylactic NPWT and overall complications (Risk Ratio (RR) 0.42; *P* < 0.001) and SSIs (RR 0.45; *P* = 0.003). Foley *et al*. suggested that the longer interval between surgery and first wound review with NPWT device usage reported in most of the included studies, compared with standard dry dressings, may have be a confounding factor for wound infection rates. When designing this study, the aim was to reflect real-world practice rather than being overly prescriptive with how experienced surgeons should manage their postoperative patients. Through the large sample size, individual surgeon preference for the time of first dressing changes should be equitably distributed through the control group with potential for a *post hoc* subanalysis.

Furthermore, this randomized feasibility trial aims to evaluate the cost-effectiveness, tolerability, and impact on patients’ quality of life of NPWT. In their Cochrane review of NPWT across surgical specialities, Normal *et al*.^19^ noted that pain and health-related quality of life were not reported by most of the included RCTs. The absence of robust cost-effectiveness data to support the routine use of NPWT is also of concern. NPWT is considerably more expensive than traditional dressings (approximately £120–145 versus £4 for a standard dressing), and cost-effectiveness studies^19^ produced differing results across varied settings with no high-grade evidence of cost efficacy. While comparing the cost of the device against the potential saved cost of avoided complications, the cost of devices used improperly without achieving the desired effect must also be considered. As such, it is felt that the feasibility outcome of monitoring the durability of these devices in the ward setting on amputation stumps will provide helpful information. Potential pitfalls in real-world practice using NPWT devices include premature removal by unfamiliar healthcare staff or devices left unsealed where the colloid interface soaks with exudate and macerates the wound edges. The anticipated strength of this trial is its pragmatic design and adequate power to account for the multiple confounding factors associated with poor wound healing in this high-risk cohort.

## Supplementary Material

zraf159_Supplementary_Data

## Data Availability

Not applicable for protocol. Pseudoanonymized trial results will be available on request for independent verification for a period of up to 10 years, as accounted for in ethics approval.
