# Supplementary material for: Prophylactic Incisional Negative Pressure wound therapy (NPWT) for major Amputations (PINTA): protocol for randomized controlled trial of single-use NPWT devices for closed-incision major lower extremity amputations
Source: BJS Open. 2026 Jan 1;10(1):zraf159. doi: 10.1093/bjsopen/zraf159 (PMC12781197; doi:10.1093/bjsopen/zraf159)
Supplement: zraf159_Supplementary_Data [file zraf159_supplementary_data.docx]

**Title:** Prophylactic Incisional Negative pressure Wound Therapy (NPWT) for major Amputations (PINTA): protocol for randomised controlled trial of single-use negative pressure wound therapy devices for closed incision major lower extremity amputations

**Authors:** Megan Power Foley^1,2^, Ciara Fahey^3^, Anne-Marie Byrne^3^, Roisín Leahy^3^, Laura Dempsey^3^, Daniel Westby^1^, Stewart R Walsh^1,3,4^

**Author Affiliations:**

1. University College Hospital Galway, Newcastle Road, Galway, Ireland
2. Irish Surgical Research Collaborative, Dublin, Ireland
3. National Surgical Research Support Centre, Royal College of Surgeons Ireland, Dublin, Ireland
4. Lambe Institute for Translational Research, University of Galway, Galway, Ireland

**Corresponding Author:**

Megan Power Foley

Address: Royal College of Surgeons in Ireland, Dublin, Ireland

Telephone: +353 87 1312557

Email: [meganpfoley@rcsi.com](mailto:meganpfoley@rcsi.com)

**ORCID ID:** [**0000-0002-7731-3182**](https://orcid.org/0000-0002-7731-3182)

**Twitter handle: @meganpowerfoley @ISRCtweets @RCSI_NSRSC**

**Supplementary Materials - Index**

| **Supplementary Appendixes** |  |
| --- | --- |
| Patient Information Leaflet | *pag. 2* |
| Ethics Approval Letter | *pag. 8* |
|  |  |
|  |  |

**Patient Information Leaflet**

| **Study Title:** PINTA: Prophylactic Incisional Negative Pressure Wound Therapy (NPWT) for Amputations - A randomised controlled feasibility trial comparing single-use negative pressure wound therapy device to standard wound management in patients following closed incision major lower limb amputation |
| --- |

**Principal investigator’s name:** Stewart Redmond Walsh

**Principal investigator’s title:** Professor of Vascular Surgery

**Telephone number of principal investigator:** 091 524 222

**Data Controller’s/joint Controller’s Identity:** Ms Megan Power Foley

**Data Controller’s/joint Controller’s Contact Details:** [megan.powerfoley@hse.ie](mailto:megan.powerfoley@hse.ie) / 0871312557

**Data Protection Officer’s Identity:** Prof Stewart Walsh

**Data Protection Officer’s Contact Details:**  [stewartredmond.walsh@nuigalway.ie](mailto:stewartredmond.walsh@nuigalway.ie) / 091 524 222

You are being invited to take part in a research study to be carried out at University College Hospital Galway by Professor Stewart Walsh.

Before you decide whether or not you wish to take part, you should read the information provided below carefully and, if you wish, discuss it with your family, friends or GP (doctor). Take time to ask questions – don’t feel rushed and don’t feel under pressure to make a quick decision.

You should clearly understand the risks and benefits of taking part in this study so that you can make a decision that is right for you. This process is known as ‘Informed Consent’.

You don't have to take part in this study. If you decide not to take part it won’t affect your future medical care.

You can change your mind about taking part in the study any time you like.  Even if the study has started, you can still opt out.  You don't have to give us a reason.  If you do opt out, rest assured it won't affect the quality of treatment you get in the future.

| **Why is this study being done?** |
| --- |

Amputation is a surgical procedure performed for patients whose leg cannot be saved. Common reasons for leg amputation include severe infection, extensive damage to muscles and bones that renders the leg poorly unfunctional or gangrene (irreversible tissue death) from lack of blood supply. The level of amputation can be either below or above the knee. Patients undergoing this operation are at a risk of wound breakdown and progressive infection or tissue death, which can lead to prolonged hospital stays, delayed rehabilitation efforts and further amputation. As the number of Irish adults with diabetes rises, the number of patients undergoing amputations for complications of diabetes will increase, as will the costs of managing complex foot care. As such, identifying how we can improve patient outcomes after this procedure is an important research priority.

Research to date has focused the patient-related factors that contribute to poor healing, including poor kidney function, poor diabetic control, poor blood supply to the foot, cigarette smoking and obesity. However, there is minimal research on the impact of different types of surgical dressings on wound healing after amputation. Current practice involves using dry, adhesive dressings and protective padding. There is a large body of evidence from other surgical cuts, including sternotomies (open heart surgery) and laparotomies (surgery in the abdomen), that single-use negative pressure wound therapy (NPWT) devices reduce infection rates and promote wound healing. There is preliminary evidence from other trials like this study that these NPWT devices also improve wound healing after leg amputation. This research study is taking place to find out if the NPWT dressing is associated with fewer wound complications, faster healing and fewer re-operations after leg amputations.

| **Who is organising and funding this study?** |
| --- |

This study is organised by Professor Stewart Walsh in collaboration with National University of Ireland Galway and the Royal College of Surgeons in Ireland. Research funding will be provided by the National Surgical Research Support Centre at RCSI. At present, no grant applications have been submitted. This study is not affiliated with trial investigators obtaining a post-graduate qualification. No pharmaceutical companies have provided funding or sponsorship for this study. None of the trial investigators will receive financial compensation for recruiting patients to this study.

| **Why am I being asked to take part?** |
| --- |

You are being asked to take part in this study as you are undergoing a leg amputation at one of our trial centres.

| **How will the study be carried out?** |
| --- |

This study will take place between July 2023 and July 2025. Patients undergoing leg amputation at any of the participating trial centres will be considered for recruitment in the study. All surgeries will take place in a designated trial centre. We anticipate 820 people will participate in the study.

Patients who participate in the study will undergo their operation (leg amputation). The dressing on the surgical cut at the end of the operation will be randomly selected by a computer programme and the rest of the operation will be at the discretion of the operating surgeon. As part of the trial, patients will get a photograph of their surgical cut taken at 30 days after the operation to assess wound healing and again at 6 weeks. Furthermore, patients will be asked to rate their quality of life and out-of-pocket expenses at 30 days and 6 weeks after surgery.

| **What will happen to me if I agree to take part?** |
| --- |

If you participate in this study, you will have your leg amputation as planned. A computer programme will randomly choose what kind of dressing your surgeon applied to your leg at the end of the surgery. Your surgeons would be unlikely to discuss this detail of your operation with you in normal circumstances. Besides the choice of dressing, the trial will have no impact on your surgery, which will be performed in the normal fashion. The trial will not impact what kind of anaesthetic you receive for your surgery. You will not require any additional or experimental procedures as part of the trial. The rest of your care after surgery, i.e. duration of antibiotics, will be unrelated to the trial and will be decided by your consultant based on your individual needs. You will not need to be in hospital for a longer duration because of the trial. After you are discharged from hospital, your wound will be managed by your local public health nurses, as is the standard of care.

You will have two trial-mandated follow-up appointments after surgery, the first at 30 days and the second at six weeks. These appointments may be in person at the hospital where you had your surgery or by telephone, depending on whether your wound had already healed by the time you left the hospital. All patients who undergo an operation are routinely seen in clinic, as such these visits are not to be unexpected. Insofar as possible, we will try coordinate your routine follow-up with trial follow-up to avoid inconvenient, supernumerary clinic appointments. At 30 days and six weeks, we will take an anonymised picture of your surgical wound. We will also ask you to rate your quality of life in five main domains using a questionnaire; the trial investigator will record your answers, you will not have to fill in any documents yourself. The question should take approximately three minutes to fill out. Your answers will not be shared without anyone outside the trial. The same process will be done for an out-of-pockets expenses survey.

The research team will view your medical records to record specific details relevant to the trial; they will not be looking at aspects of your medical history unrelated to the trial. Throughout your participation in the trial, every effort will be made to keep your medical information private.

| **Video/and or Audio recordings?** |
| --- |

There will be no video or audio recordings made during this trial.

| **What other treatments are available to me?** |
| --- |

If you are being considered for this study, your consultant surgeon has already discussed treatment options with you and decided that an amputation is in your best interests.

If you do not participate in the trial, your amputation will go ahead as planned. If you consent to participate in this study but ultimately do not undergo an amputation, you will not be included.

| **What are the benefits?** |
| --- |

There are no added benefits to participating in the trial. Participation will not prioritise the timing of your surgery. There is no guarantee that one wound dressing is better than the other. However, future patients undergoing leg amputations will hopefully benefit from the information gathered by this trial, and your participation in it.

| **What are the risks?** |
| --- |

The standard risks associated with leg amputation all apply, such as bleeding, pain, infection, wound breakdown, impaired balance and cosmetic appearances, further necrosis and the need for more proximal amputation. There are no new surgical risks associated with participating in the trial, as the procedure will be carried out in the standard fashion.

There is always a potential risk for confidentially breaches if the data stored in our secure server is compromised.

| **What if something goes wrong when I’m taking part in this study?** |
| --- |

The standard supports offered to all patients undergoing leg amputation will be available to patients participating in the trial, including physiotherapy, occupational therapy, medical social worker, rehabilitation medicine, as well as access to the vascular surgery outpatients clinic.

As the trial does not involve an experimental procedures or advanced diagnostics, we do not anticipate participate in the trial will lead to additional distress or discovery of unexpected pathology.

We advised any patients undergoing leg amputation, regardless of trial participation, to present to their GP if they feel unwell at home after hospital discharge.

| **Will it cost me anything to take part?** |
| --- |

Participating in the trial should not incur any additional costs to you compared to undergoing leg amputation without trial participation.

| **Is the study confidential?** |
| --- |

The trial investigators will endeavour to keep your patient information private and confidential. The information collected for this study will be anonymised with each participant assigned a unique identification number. You will not be identifiable from this information. In the pilot site, the data will be kept on a desktop computer in an encrypted, password-protected excel file; only the trial investigators will have password access. Should the trial expand to multiple sites, the data will be stored in a secure computer database (REDCap).

It is highly unlikely that the trial investigators will need to contact your GP about the trial specifically; however, your surgical team will always be available to your GP if they have ongoing concerns about your surgical wound. The trial investigators will collect pre-designated baseline data from your medical records and note the results of your follow-up care; no further information will be accessed.

The information will be kept until the feasibility study, and any successive randomised controlled trials, are completed, reported and published in a scientific journal. It is our intention to publish the anonymised results of the trial in the medical literature and present them at medical conferences with the intention of improving patient care. You will not be identified in any publication. At this point, any photographs used to track wound progress will be destroyed. No further tests will be performed specifically for trial purposes, as such there will be no more personal results to share. If you are interested, we will circulate the final results of the entire study to you.

| **Data Protection** |
| --- |

1. We will be using your personal information in our research to help us study wound healing after limb amputation;
2. The legal basis under your data is being processing for the purpose of scientific research;
3. The trial investigators are only people who will have access to your data collected for the purpose of this trial;
4. The data will be stored for ten years, as is the requirement with randomised controlled trials. This period is to facilitate third-party verification of trial results, should they questioned;
5. There is a risk that your data could be shared or stolen, and your confidentiality could be breached. Release of this data could inadvertently cause you harm;
6. You have the right to withdraw your consent for your data to be used in this trial at any stage. Should you wish to withdraw, contact Prof. Stewart Walsh at [stewartredmond.walsh@nuigalway.ie](mailto:stewartredmond.walsh@nuigalway.ie);
7. You have a right to lodge a complaint with the Data Protection Commissioner;
8. You have right to request access to your data and to receive a copy of it, unless your request renders it impossible or very difficult to conduct this research;
9. You have a right to restrict or object to processing of your data, unless your request renders it impossible or very difficult to conduct this research e.g. you don’t want your data shared but don’t mind having it collected and stored.
10. You have a right for any inaccurate information about you to corrected or deleted, unless this request would make it impossible or make it very difficult to conduct the research;
11. You have a right to have your personal data deleted, unless your request renders it impossible or very difficult to conduct the research. e.g. you want to delete your data at the end of a research project just before it is due to be published;
12. You have a right to data portability, meaning you have a right to move your data from one data controller to another in a readable format;
13. There will be no automated decision making based on your personal data in this trial;
14. You a right to object to automated processing including profiling if you wish;

1. We do not intend to further process your personal data for other purposes;
2. Your data will not be transported out of Ireland.

| **Where can I get further information?** |
| --- |

If you have any further questions about the study or if you want to opt out of the study, you can rest assured it won't affect the quality of treatment you get in the future.

If you need any further information now or at any time in the future, please contact:

Name: Prof Stewart Walsh

Address: University College Hospital Galway

Phone No: 091 524 222

**
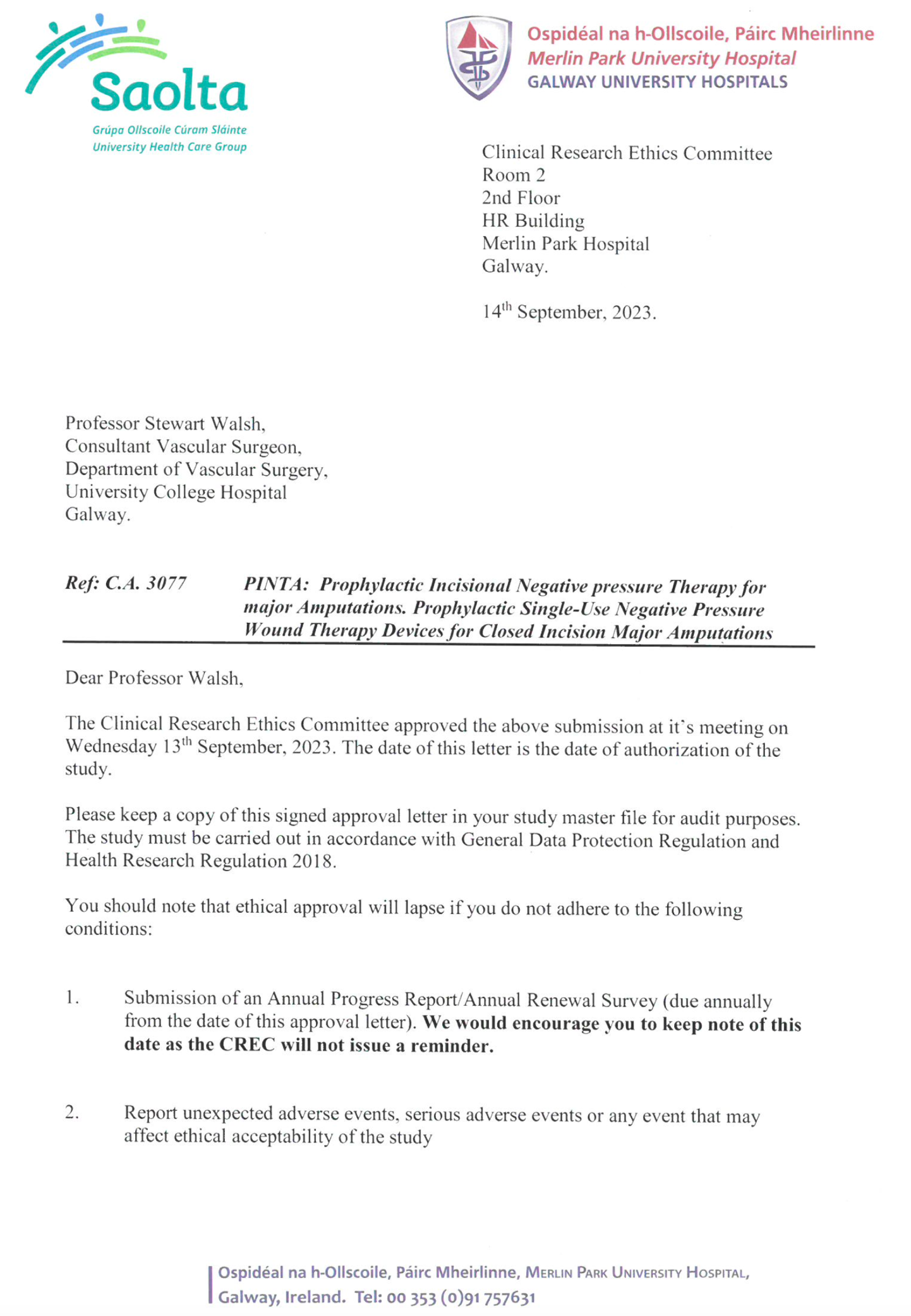
**

**
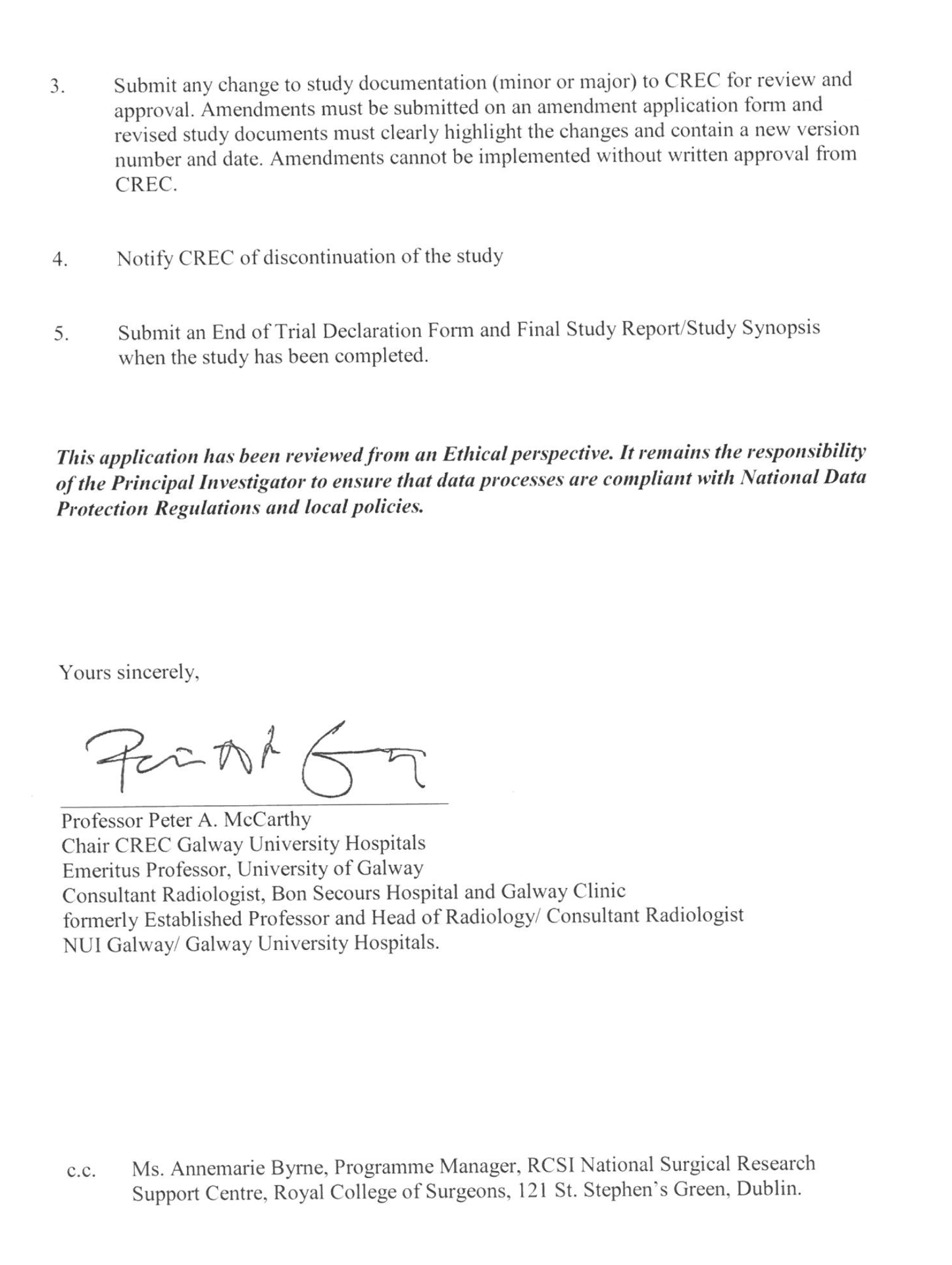
**
